# Safety and immunogenicity of Vi-typhoid conjugate vaccine co-administration with routine 9-month vaccination in Burkina Faso: A randomized controlled phase 2 trial

**DOI:** 10.1016/j.ijid.2021.05.061

**Published:** 2021-07

**Authors:** Sodiomon B. Sirima, Alphonse Ouedraogo, Nouhoun Barry, Mohamadou Siribie, Alfred Tiono, Issa Nébié, Amadou Konaté, Gloria Damoaliga Berges, Amidou Diarra, Moussa Ouedraogo, Edith C. Bougouma, Issiaka Soulama, Alimatou Hema, Shrimati Datta, Yuanyuan Liang, Elizabeth T. Rotrosen, J. Kathleen Tracy, Leslie P. Jamka, Jennifer J. Oshinsky, Marcela F. Pasetti, Kathleen M. Neuzil, Matthew B. Laurens

**Affiliations:** a Groupe de Recherche Action en Santé, Ouagadougou, Burkina Faso; b Center for Vaccine Development and Global Health, University of Maryland School of Medicine, Baltimore, MD, USA

**Keywords:** Typhoid conjugate vaccine, Typhoid fever, Burkina Faso, Yellow fever vaccine, Coadministration

## Abstract

**Objectives:**

In 2017, the World Health Organisation (WHO) pre-qualified a single-dose typhoid conjugate vaccine (TCV) and identified TCV co-administration studies as a research priority. Accordingly, we tested co-administration of Typbar TCV® (Bharat Biotech International) with measles-rubella (MR) and yellow fever (YF) vaccines.

**Methods:**

We conducted a randomized, double-blind, and controlled, phase 2 trial in Ouagadougou, Burkina Faso. Healthy children aged 9–11 months were randomized 1:1 to receive TCV (Group 1) or control vaccine (inactivated polio vaccine (IPV), Group 2). Vaccines were administered intramuscularly with routine MR and YF vaccines. Safety was assessed by (1) local and systemic reactions on days 0, 3, and 7; (2) unsolicited adverse events within 28 days; and (3) serious adverse events (SAEs) within six months after immunization.

**Results:**

We enrolled, randomized, and vaccinated 100 eligible children (49 Group 1 and 51 Group 2). Safety outcomes occurred with similar frequency in both groups: local/solicited reactions (Group 1: 1/49, Group 2: 3/50), systemic/solicited reactions (Group 1: 4/49, Group 2: 9/50), unsolicited adverse events (Group 1: 26/49, Group 2: 33/51), and SAEs (Group 1: 2/49, Group 2: 3/51). TCV conferred robust immunogenicity without interference with MR or YF vaccines.

**Conclusion:**

TCV can be safely co-administered with MR and YF vaccines to children at the 9-month vaccination visit.

## Highlights

• This is the first study on the co-administration of typhoid conjugate vaccine (TCV) in West Africa.
• Co-administration of TCV with routine vaccines in a typhoid-endemic country was successful.
• TCV was safely co-administered at nine months with yellow fever and measles-rubella vaccines.
• Single-dose TCV was immunogenic in 9-month-old children.
• There was no safety signal related to TCV vaccination or co-administration.

## Introduction

Typhoid fever, a potentially life-threatening infection caused by the bacterium *Salmonella enterica* serovar Typhi (*S*. Typhi), is spread through contaminated food and water. It is estimated that more than nine million cases of typhoid fever and more than 110,000 deaths occur worldwide each year (Institute for Health Metrics and Evaluation, 2020). Multidrug-resistant typhoid fever appeared in the 1970s and has spread globally, with 47% of *S*. Typhi isolates reported to be resistant to first-line agents (Marks et al., 2017, Wong et al., 2015).

In endemic areas, typhoid fever incidence has traditionally been highest in school-age children (5–19 years), yet is increasingly recognized as a public health problem in children under five (GBD 2019 Diseases and Injuries Collaborators, 2020). Estimates of annual incidence in sub-Saharan Africa range from 0 to 383 cases per 100,000 people, 2–3 times higher than previous estimates, with the highest incidence in children 2–14 years old (Marks et al., 2017). In Burkina Faso, estimates range from 104 to 383 cases per 100,000 person-years of observation (PYO) (Marks et al., 2017).

Vaccine uptake of earlier typhoid vaccines has been low in endemic countries, in part because none are approved for children under two years of age or subsidized by Gavi, the Vaccine Alliance (World Health Organization, 2017). In 2017, the World Health Organisation (WHO) pre-qualified the first typhoid conjugate vaccine (Typbar TCV®). (World Health Organization, 2018); TCV holds promise for typhoid fever control in Africa because of its favorable safety profile, one dose schedule, and approval for use in infants as young as six months of age, making it feasible for incorporation into routine immunization schedules.

The WHO prioritized co-administration of TCV with other childhood vaccines in its 2018 position paper (World Health Organization, 2018). Measles and yellow fever are common in many parts of the world and can rapidly spread in densely populated areas, especially among unvaccinated people. Due to increasing numbers of cases and recent global outbreaks, including several large measles outbreaks in Africa (World Health Organization, 2019), it is important to assess co-administration of any new vaccine with the measles-rubella (MR) and yellow fever (YF) vaccines to confirm no interference with immune response (Capeding et al., 2020). Burkina Faso is an ideal site for this study given its relatively high incidence of typhoid fever, high infant mortality, low prevalence of human immunodeficiency virus, and ongoing typhoid surveillance. Furthermore, the Burkina Faso immunization schedule includes MR and YF vaccines at nine months.

This study is part of the Typhoid Vaccine Acceleration Consortium (TyVAC), which aims to generate evidence to support TCV introduction as part of an integrated approach to reduce the burden of typhoid fever in endemic countries. In this study, we assessed TCV safety, immunogenicity, and non-interference with MR and YF co-administration, as well as immunogenicity to the tetanus toxoid in 9-month-old children. This study provides safety and immunogenicity data on co-administration of these routine vaccinations to inform large-scale uptake in sub-Saharan Africa.

## Methods

### Study design

We conducted a randomized, double-blind, controlled, phase 2 trial, evaluating the safety and immunogenicity of TCV when co-administered with vaccinations routinely given at 9 months of age in Burkina Faso. The study was conducted by the Groupe de Recherche Action en Santé (GRAS) at Schiphra Protestant Hospital outpatient pediatric clinic, an urban hospital in Ouagadougou, Burkina Faso, December 2018 to August 2019. The study protocol was previously published (Laurens et al., 2019).

### Participants

We enrolled children aged nine through eleven months at their routine 9-month vaccination visit. Upon arrival, groups of parents and guardians were given general information about the study. The GRAS team provided information orally (in French and local languages) and answered preliminary questions. Interested parties signed informed consent forms, and children were screened for eligibility. Children were deemed eligible if healthy and likely to remain in the study area for the trial duration. Participants were temporarily excluded for 48 h if they presented with fever or a history of fever within the previous 24 h. A complete list of inclusion and exclusion criteria is available in the study protocol (Laurens et al., 2019).

### Randomization and masking

Participants were randomly assigned to receive TCV or inactivated polio vaccine (IPV) in a 1:1 ratio. Using a blocked randomization procedure with random block sizes of six, nine, or twelve, the randomization sequence was computer-generated. After study clinicians completed screening and eligibility procedures, participants were assigned a unique treatment code generated by the study biostatistician and programmed into the Research Electronic Data Capture (REDCap) study database. The randomization module was accessible only to the unblinded study pharmacist responsible for vaccine preparation. Unblinded nurses were responsible for vaccine administration. These unblinded personnel were not involved in study-related assessments and did not have contact with participants after vaccination. In addition, investigators responsible for data analyses were unblinded.

### Procedures

Based on their randomized treatment assignment, children received one intramuscular injection of 0∙5 mL of TCV or IPV on study day 0. The TCV was developed by Bharat Biotech International, Hyderabad, India. It consists of 25 μg of Vi polysaccharide conjugated to a tetanus toxoid protein carrier. The control vaccine, IPV, is manufactured by IMOVAX POLIO, Sanofi Pasteur, Lyon France and is composed of 40 D-antigen units (DU) of poliovirus type 1, 8 DU of poliovirus type 2, and 32 DU of poliovirus type 3 preserved in 2-phenoxyethanol (5 mg/mL). The study pharmacist prepared the TCV and IPV in individual syringes behind a privacy screen, so parents/guardians remained blinded to study treatment. On study day 0, all participants received the MR vaccine subcutaneously in the right deltoid, the YF vaccine subcutaneously in the left deltoid, and either TCV (Group 1) or IPV (Group 2) intramuscularly in the left thigh.

To document adverse events following immunization, children were observed at the clinic for 30 min post-vaccination on day 0. Follow-up clinic visits were scheduled at three, seven, 28 and 180 days after vaccination. A solicited vaccine reactogenicity assessment was performed on day three, collecting post-vaccination information from days 0 and three, and seven. Unsolicited adverse events were captured for 28 days after vaccination, and serious adverse events (SAEs) were captured for 180 days throughout study follow-up. On day 28, 3–5 mL of blood was drawn to measure immune responses. Unscheduled visits followed standard procedures for medical evaluation and treatment established by the Burkina Faso Ministry of Health. Illness and other untoward medical events were documented as adverse events in participant records. The seriousness, severity, relationship to the study product, and expectedness were recorded in accordance with the United States Food and Drug Administration’s (FDA) guidelines for vaccine clinical trials (Dimonte et al., 2016). Clinical data were directly entered into the electronic case report form in REDCap.

Anti-Vi serum IgG antibody levels were measured by enzyme-linked immunosorbent assay (ELISA) using a commercial kit (VaccZyme, The Binding Site Group Ltd., Birmingham, UK). Anti-tetanus toxoid serum IgG antibody levels were quantified using an ELISA kit (EuroImmun AG, Luebek, Germany). Both anti-Vi IgG and anti-tetanus antibody testing were done at the Groupe de Recherche Action en Santé (GRAS) laboratories in Ouagadougou, Burkina Faso. The Walter Reed Army Institute of Research in Silver Spring, Maryland, USA, conducted the Plaque Reduction Neutralization Test (PRNT) to measure the ability of yellow fever-specific antibodies to neutralize the macroscopic cytopathology caused by a single virus particle on a monolayer of Vero cells (Mattiuzzo et al., 2019). Titers are reported in milli international units per milliliter (mIU/mL) according to the National Institute for Biological Standards and Control Reference Standard: 15,790 (Reference MNID-0952). Serum anti-measles and anti-rubella antibodies were measured by ELISA at the Center for Vaccine Development and Global Health, University of Maryland School of Medicine, Baltimore, Maryland, USA, using standard methods (Simon et al., 2011; Tapia et al., 2005). For measles, plates were coated with measles virus lysate (Advanced Biotechnologies, Inc. Columbia, MD) at 5 μg/mL, and the anti-measles WHO 3rd International Standard (NIBSC 97/648) to report titers in mIU/mL. For rubella, plates were coated with 0∙5 μg/mL of rubella K1S antigen (Microbix Biosystems Inc. #EL-05-10), and the anti-rubella immunoglobulin WHO International Standard (NIBSC RUBI-1-94) was used as standard with titers being reported in IU/mL. We assessed baseline *Plasmodium falciparum* co-infection by microscopic evaluation of a thick blood smear collected on day 0. Blood smears were examined at GRAS laboratories in Ouagadougou according to standard procedures by two independent technicians with documented expertise in malaria microscopy.

### Outcomes

The primary objective was to assess TCV safety when co-administered with Expanded Programme on Immunisation (EPI) vaccines among children nine through eleven months of age in Burkina Faso. The primary safety outcomes were the proportion of participants who experienced: (1) adverse events in the first 30 min after vaccination and seven days following vaccinations, (2) other non-serious adverse events up to 28 days after vaccination, and (3) other SAEs up to six months following vaccination.

Secondary outcomes included (1) YF immunogenicity with and without TCV and (2) TCV immunogenicity when given with YF. In addition, seroconversion was determined by comparing antibody levels from days 0 and 28; participants who attained a ≥4-fold rise in anti-Vi antibody titers were considered protected against typhoid fever, and those who attained anti-YF IgG level ≥500 mIU/mL were considered protected against yellow fever.

Exploratory immunogenicity outcomes included tetanus toxoid IgG antibody levels and MR vaccine immunogenicity when given with and without TCV. Participants with anti-tetanus IgG levels of 0·1 to <1·0 IU/mL were considered to have short-term immunity, while those with levels ≥1·0 IU/mL were considered to have long-term immunity. Children with anti-measles IgG level ≥120 mIU/mL and anti-rubella IgG level ≥10 IU/mL were considered seroprotected.

### Statistical analysis

The sample size was calculated using PASS 15 based on the null hypothesis that when a single dose of TCV (Group 1) or IPV (Group 2) is given with YF and MR, reactogenicity is not significantly different. Therefore, the expected incidence of any grade 2 or higher solicited, systemic adverse event within seven days following vaccination is 10% in Group 2. (Haidara et al., 2018). A sample size of 45 participants in each group achieves 80% power to detect a minimum difference of 23% (33% minus 10%), using a 2-sided z-test with unpooled variance and a significance level of 5%.

The distribution of each antibody measure of interest at each time point was examined graphically and described in terms of sample size, geometric mean, and corresponding 95% confidence intervals. When computing geometric means, zeros or those values that were below the limit of detection were replaced by one half the limit of detection (i.e., 3∙7 EU/mL for anti-Vi, 45 mIU/mL for anti-yellow fever, 0∙85 mIU/mL for anti-measles, and 0∙05 IU/mL for anti-rubella). Antibody values were log10 transformed. The transformed data were compared between the TCV and IPV groups using a two-sample t-test; and were compared between day 0 and day 28 within each group using a paired t-test. For each antibody of interest, seropositivity rate was computed at each time point for each group and compared between groups using Fisher’s exact test.

All participants who received TCV or IPV were included in the safety analysis, and participants who received all assigned vaccinations were included in the per-protocol analysis. Study results were analyzed using SAS software, Version 9·4, SAS System for Windows (Copyright® 2016 SAS Institute Inc.). All vaccines in this study are approved and pre-qualified by the WHO; therefore, no Data and Safety Monitoring Board oversaw the study. The trial is registered at ClinicalTrials.gov, Identifier NCT03614533.

## Findings

**Figure 1:**
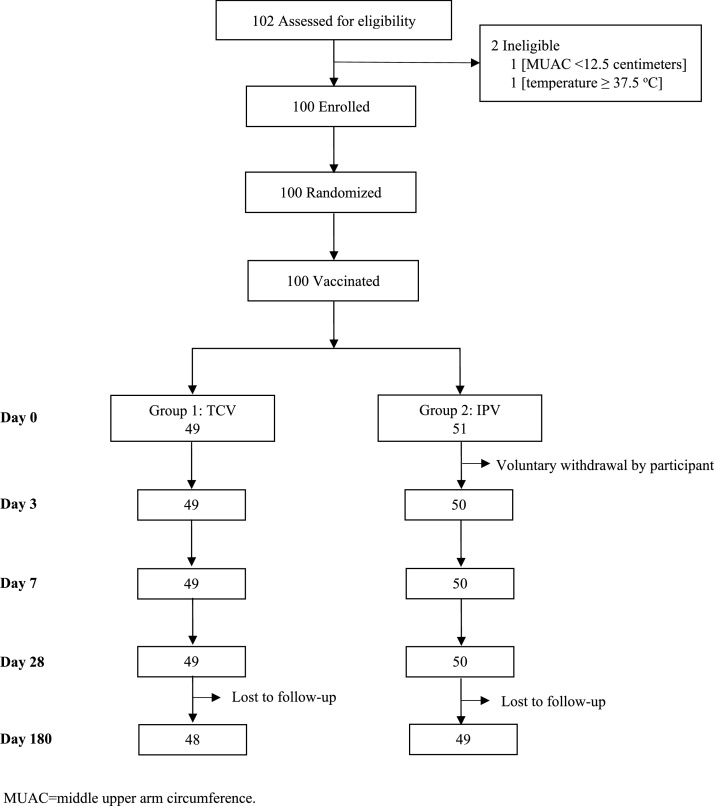
Disposition of participants (CONSORT flow diagram).

The study was conducted from December 03, 2018 to August 25, 2019. The consort diagram (Figure 1) shows the disposition of participants. A total of 102 children were screened; of these, 100 (51 females and 49 males) were eligible, enrolled, and vaccinated. Forty-nine participants received TCV, and 51 received IPV. A total of 99 participants completed days three, seven, and 28 visits (one child withdrew after vaccination), and 97 completed their 6-month follow-up to ascertain adverse events. Two participants did not attend the day 180 visit and were considered lost to follow-up.

Study groups were comparable in baseline demography, height, weight, middle upper arm circumference (MUAC), and baseline immunogenicity measurements (Table 1). Most participants were approximately nine months of age. Baseline immunity against measles, rubella, tetanus, and yellow fever were similar in both groups.

**Table 1:** Demographics and baseline characteristics of study participants at enrolment.

	Group 1: TCV	Group 2: IPV
Enrolled (n = 100)	49	51
Vaccinated (n = 100)	49	51
Sex		
Female (n = 51)	25 (51·0%)	26 (51·0%)
Male (n = 49)	24 (49·0%)	25 (49·0%)
Age in months	9·7 (0·8)	9·4 (0·4)
Weight in kilograms	8·4 (1·1)	8·3 (1·1)
Height in centimeters	70·4 (4·0)	69·6 (4·2)
Middle upper arm circumference (MUAC) in centimeters	14·2 (1·2)	14·0 (0·9)
Number of participants with baseline Vi titer (≥7·4 EU/mL)	18 (36·7%)	15 (29·4%)
Number of participants with baseline seropositive measles titer (≥120 mIU/mL)	0 (0%)	1 (2·0%)^a^
Number of participants with baseline seropositive rubella titer (≥10 IU/mL)	4 (8·2%)	8 (16·0%)^a^
Number of participants with baseline tetanus titer (≥0·001 IU/mL)	49 (100·0%)	51 (100·0%)
Number of participants with baseline yellow fever titer (≥90 mIU/mL)	0 (0·0%)	1 (2·0%)^a^
Number of participants with baseline malaria positivity	1 (2·0%)	0 (0·0%)

Data are n (%) or mean (SD).

n = number of participants, SD = standard deviation, EU = ELISA units, IU = international units, TCV = typhoid conjugate vaccine, IPV = inactivated poliovirus vaccine.

a One participant missing day 0 titre, n = 50.

Solicited reactogenicity was infrequent among both groups. Four participants (4/99 = 4%) experienced local symptoms consisting of swelling and pain or tenderness within seven days after vaccination. One local reaction occurred in the IPV group on day 0 (1/50 = 2·0%, 95%CI 0·1–10·7%) and two on day seven (2/50 = 4·0%, 95%CI 0·5–13·7%), and one local reaction occurred in the TCV group on day three (1/49 = 2·0%, 95%CI 0·0%–11·7%), all assessed as mild. Thirteen (13/99 = 13%) participants experienced mild or moderate systemic reactogenicity, including fever or feverishness and irritability (Table 2). No severe solicited symptoms were observed. Of the 59 non-serious adverse events, all were mild or moderate and none associated with the study vaccine. No adverse events were observed in the first 30 min after vaccination. Adverse event frequency was similar among groups, and over 50% of participants in each study group experienced at least one adverse event during the 28 days after vaccination; common events included upper respiratory tract infections, diarrhea, and fever. Two participants in the TCV group and three in the IPV group experienced six SAEs, and one participant in the TCV group experienced two SAEs. All SAEs were not related to the study vaccine and resolved without sequelae (Table 2).

**Table 2:** Summary of safety parameters by group, participant level.

Systemic reactions		Group 1: TCV (N = 49)		Group 2: IPV (N = 51)	
		n	% (95% CI)	n	% (95% CI)
Day 0^a^	Any systemic reaction	2	4·1 (0·5–14·0)	6	12·0 (4·5–24·3)
	Fever	1	2·0 (0·1–10·9)	4	8·0 (2·2–19·2)
	Irritability	1	2·0 (0·1–10·9)	2	4·0 (0·5–13·7)

Day 3^a^	Any systemic reaction	1	2·0 (0·1–10·9)	3	6·0 (1·3–16·6)
	Fever	1	2·0 (0·1–10·9)	2	4·0 (0·5–13·7)
	Irritability	1	2·0 (0·1–10·9)	1	2·0 (0·1–10·7)

Day 7^a^	Any systemic reaction	1	2·0 (0·1–10·9)	3	6·0 (1·3–16·6)
	Fever	0	0·0 (0·0–7·3)	2	4·0 (0·5–13·7)
	Irritability	1	2·0 (0·1–10·9)	1	2·0 (0·1–10·7)

Days 0, 3, or 7	Any systemic reaction	4	8·2 (2·3–19·6)	9	18·0 (8·6–31·4)

Adverse events, unsolicited					
	Any adverse event	26	53·1 (38·3–67·5)	33	64·7 (50·1–77·6)
	Conjunctivitis	2	4·1 (0·5–14·0)	3	5·9 (1·2–16·2)
	Cough	0	0·0 (0·0–7·3)	5	9·8 (3·3–21·4)
	Diarrhea	7	14·3 (5·9–27·2)	11	21·6 (11·3–35·3)
	Fever with no source	7	14·3 (5·9–27·2)	9	17·7 (8·4–30·9)
	Malaria	1	2·0 (0·1–10·9)	2	3·9 (0·5–13·5)
	Other rash or skin disorder	3	6·1 (1·3–16·7)	5	9·8 (3·3–21·4)
	Upper respiratory illness	15	30·6 (18·3–45·4)	17	33·3 (20·8–47·9)
	Vomiting	4	8·2 (2·3–19·6)	3	5·9 (1·2–16·2)
	Other	2	4·1 (0·5–14·0)	3	5·9 (1·2–16·2)

Serious adverse events					
	Any serious adverse event	2	4·1 (0·5–14·0)	3	5·9 (1·2–16·2)
	Diarrhea/gastroenteritis	1	2·0 (0·1–10·9)	1	2·0 (0·1–10·5)
	Malaria	1	2·0 (0·1–10·9)	0	0·0 (0·0–7·0)
	Other rash or skin disorder	0	0·0 (0·0–7·3)	1	2·0 (0·1–10·5)
	Respiratory illness	1	2·0 (0·1–10·9)	1	2·0 (0·1–10·5)

n = number of participants, CI = confidence interval.

a One participant in Group 2 missed Day3 (when Day 0 and Day 3 reactogenicity information was collected) and Day 7 visit.

**Figure 2:**
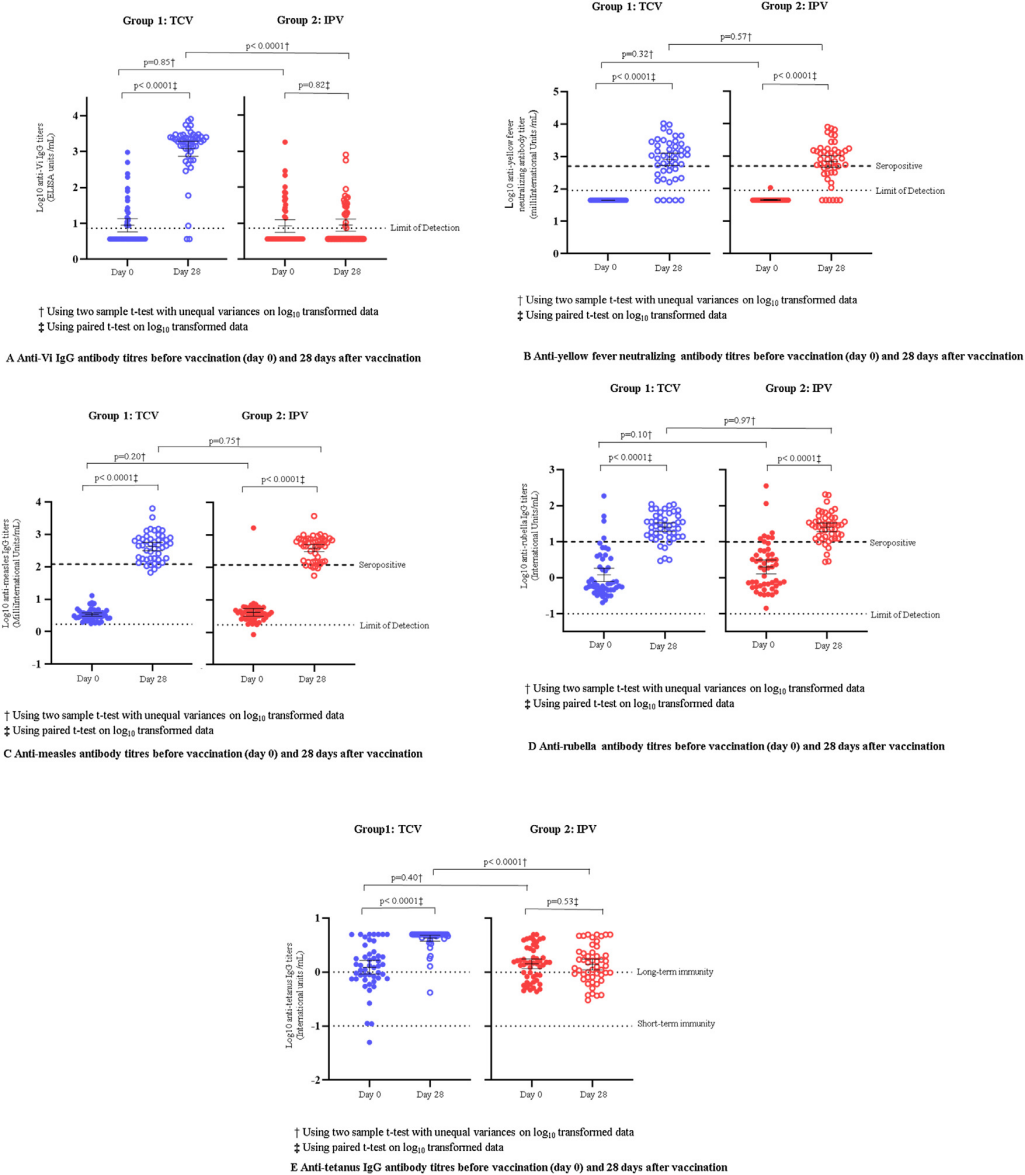
(A) Anti-Vi IgG antibody titers before vaccination (day 0) and 28 days after vaccination. (B) Anti-yellow fever neutralizing antibody titers before vaccination (day 0) and 28 days after vaccination. (C) Anti-measles titers before vaccination (day 0) and 28 days after vaccination. (D) Anti-rubella antibody titers before vaccination (day 0) and 28 days after vaccination. (E) Anti-tetanus IgG antibody titers before vaccination (day 0) and 28 days after vaccination.

On day 0, anti-Vi antibody titers were below the limit of assay detection for 36/51 (71%) of IPV recipients and 31/49 (63%) of TCV recipients and significantly higher on day 28, post-vaccination, for the TCV group (Table 3 and Figure 2A). Before vaccination, anti-Vi antibody geometric mean titer (GMT) was 8·9 and 8·5 ELISA units (EU)/mL for TCV and IPV recipients, respectively. Post-vaccination, GMT rose to 1203·7 EU/mL for the TCV group and remained relatively unchanged at 8·9 EU/mL for the IPV group. Thus, a total of 43/49 (87·8%) participants vaccinated with TCV achieved seroconversion. Analyses by gender show no significant difference between males and females that received TCV or IPV (Supplementary Appendix Table A1).

**Table 3:** Anti-Vi IgG antibody immunogenicity before vaccination (day 0) and 28 days after vaccination.

	Group 1: TCV (N = 49)	Group 2: IPV (N = 51)
Seroconversion^a^ (%, 95% CI)	43/49 (87·8%, 75·2–95·4)	4/50^b^ (8·0%, 2·2–19·2)
Day 0 geometric mean titer (95% CI)	8·9 (5·9–13·6)	8·5 (5·6–12·7)
Day 28 geometric mean titer (95% CI)	1203·7 (747·1–1939·5)	8·9^b^ (6·1–13·1)

N = total number, CI = confidence interval.

Geometric mean titer in ELISA units (EU)/mL.

a ≥4-fold rise from day 0 to 28 days after vaccination.

b One participant in the IPV group missing titer, n = 50.

Anti-yellow fever antibody titers for both groups were below the detection limit on day 0 except for one participant (1/99 = 1∙0%) in the IPV group and then measured significantly higher post-vaccination (Table 4 and Figure 2B). Anti-yellow fever antibody GMT was 45·0, and 45·8 mIU/mL for TCV and IPV groups, respectively, before vaccination. Post-vaccination, these levels rose to 815·5 mIU/mL for the TCV group and 688·5 mIU/mL for the IPV group. On day 28, 32/46 (70%) of participants in the TCV group and 31/48 (65%) in the IPV group were seropositive for anti-yellow fever antibodies at the 500 mIU/mL threshold.

**Table 4:** Anti-measles and anti-rubella IgG and yellow fever neutralizing antibody immunogenicity before vaccination (day 0) and 28 days after vaccination.

		Yellow fever			Measles			Rubella		
	N	GMT (95% CI)	Seropositivity^a^		GMT (95% CI)	Seropositivity^a^		GMT (95% CI)	Seropositivity^a^	
			n	% (95% CI)		n	% (95% CI)		n	% (95% CI)
Day 0										
Group 1: TCV	49	45·0 (45·0–45·0)	0	0·0 (0·0–7·3)	3·4 (3·0–3·8)	0	0·0 (0·0–7·3)	1·2 (0·8–1·8)	4	8·2 (2·3–19·6)
Group 2: IPV	50	45·8 (44·2–47·5)	0	0·0 (0·0–7·1)	4·1 (3·1–5·4)	1	2·0 (0·1–10·7)	2·0 (1·3–3·1)	8	16·0 (7·2–29·1)
p-Value^b^		0·32		NA	0·20		1·00	0·10		0·36
Day 28										
Group 1: TCV	46	815·5 (527·1–1261·6)	32	69·6 (54·3–82·3)	442·5 (314·9–566·8)	41	89·1 (76·4–96·4)	25·3 (19·3–33·3)	40	87·0 (73·7–95·1)
Group 2: IPV	48	688·5 (455·1–1041·5)	31	64·6 (49·5–77·8)	396·8 (307·6–511·8)	42	87·5 (74·8–95·3)	25·5 (19·4–33·5)	42	87·5 (74·8–95·3)
p-Value^b^		0·57		0·66	0·75		1·00	0·97		1·00

n = number of participants, N = total number, GMT = geometric mean titer, CI = confidence intervals, NA = not applicable, IU = international units.

Measles and yellow fever GMT are reported in mIU/mL. Rubella GMT is reported in IU/mL.

a Anti-yellow fever titer ≥500 mIU/mL; anti-measles titer ≥120 mIU/mL; anti-rubella titer ≥10 IU/mL.

b Using two-sample t-test with unequal variances on log_10_ transformed data or Fisher’s Exact test on proportions.

The anti-measles antibody GMT for both groups was low on day 0 (3·4 for TCV and 4·1 for IPV) and significantly higher post-vaccination (422·5 for TCV and 396·8 for IPV) (Table 4 and Figure 2C). The same trend for rubella antibody titers was seen in both groups (Table 4 and Figure 2D). Almost 90% of participants in both TCV and IPV groups were seropositive for anti-measles and for anti-rubella antibodies.

Anti-tetanus IgG antibody titers for both groups were above the threshold for short-term immunity post-vaccination. However, in the TCV group, 48/49 (98%) of participants were above the threshold for long-term immunity post-vaccination, whereas the number was similar pre- and post-vaccination in the IPV group (Supplementary Appendix Table A2 and Figure 2E).

## Discussion

Data on co-administration with routine vaccinations are imperative for informing decisions to add a new vaccine to the existing immunization regimen. For this reason, the WHO prioritized studies of TCV co-administration with routine childhood vaccines. The primary goal of this study was to ensure TCV would not adversely impact the safety and immunogenicity of lifesaving vaccines that Burkinabe children currently receive. In this cohort, TCV was well-tolerated and did not interfere with the immune response to co-administered vaccines. Before this study of TCV in Burkina Faso, no co-administration data for TCV with MR and YF vaccines were available.

This safety and reactogenicity profile documented in Burkinabe infants is consistent with studies of this TCV from Burkina Faso and other parts of the world. (April 04–6, 2017, Milligan et al., 2018, Mohan et al., 2015, Qamar et al., 2020, Shakya et al., 2019, Sirima et al., 2020) In our study, 4 infants that received TCV (8·2%) experienced fever and irritability in the seven days after vaccination, which is almost identical to the rate of 8·1% observed in a study of 15-month-olds in Burkina Faso (Sirima et al., 2020) and less than the observed rate of 18% for IPV recipients in the current study. As IPV is a well-accepted vaccine routinely administered to infants as young as six weeks of age, the current finding that TCV was tolerated as well as, if not better than, IPV bodes well for TCV acceptance among caregivers and healthcare practitioners.

A recent study in Burkina Faso showed young children vaccinated with TCV at the 15-month visit with both group A meningococcal conjugate vaccines and their second MR vaccine, experienced low reactogenicity and no safety concerns (Sirima et al., 2020). Few (8·1%) experienced mild fever and/or mild irritability seven days after vaccination (Sirima et al., 2020). In India, among 360 infants and children 6–23 months of age who received TCV, few experienced fever (2·4%), injection site pain (3·6%), or tenderness (0·6%) after vaccination (Mohan et al., 2015). In Hyderabad, Pakistan, among 7139 children aged six months to ten years who received TCV and were actively followed for safety, the most common adverse events reported in the 14 days after immunization included fever (2·9%) and pain or swelling at the injection site (1·9%) (Qamar et al., 2020). Similarly, a TCV study that assessed adverse reactions in the first seven days after a single dose vaccination in Nepalese infants and children ages nine months to sixteen years reported a general unwell status (6·7%), pain at the injection site (5·1%), and fever (5·0%) in TCV recipients (Shakya et al., 2019). Overall, these studies support the safety and tolerability of TCV.

TCV increased the long-term anti-tetanus antibody levels with significant seroconversion (98%). While not the primary goal of TCV immunization, this could present an added value of TCV in resource-poor settings where vaccine coverage is not optimal (Hoest et al., 2017). In Burkina Faso and many other sub-Saharan African countries, tetanus immunization is recommended at eight, twelve, and 16 weeks of age and is not subsequently boosted. Though we did not verify that our study participants previously received all three scheduled doses of the tetanus vaccine, results demonstrate that this 3-dose schedule provides a short-term tetanus immunity to most infants that is maintained at 9-months of age and long-term immunity in about 60%. The additional boost of tetanus toxoid antigen in TCV provided long-term tetanus protection in all but one TCV recipient. This long-term protection against tetanus provides evidence that TCV can have a meaningful public health impact beyond typhoid fever prevention, and this benefit should be considered when weighing the total public health value of TCV implementation. While administration of TCV with Vi polysaccharide fused to a tetanus toxoid carrier increases the cumulative exposure of children to tetanus toxoid, as above, we did not see increased reactogenicity to TCV.

A single dose of TCV demonstrated a robust antibody response in Burkinabe infants at nine months of age, including day 28 seroconversion in 87·8% with GMT of 1203·7 EU/mL. These results are consistent with previous studies of this vaccine (Jin et al., 2017, Kundu et al., 2020, Mohan et al., 2015, Shakya et al., 2019, Sirima et al., 2020) A study in Indian children ages 6 months to 2 years documented day 42 GMT of 1115 EU/mL (Kundu et al., 2020). A separate study in Indian children ages 24 months to 4 years found day 42 seroconversion in 97·3% with a GMT of 1293 EU/mL (Mohan et al., 2015). In 15-month-old Burkinabe children, the day 28 seroconversion of 94–96% was higher than the current study with a GMT of 2757–3707 EU/mL (Sirima et al., 2020). The higher responses in the 15-month-old children may reflect an enhanced immune response in children with more mature immune systems. There is no correlate of protection for typhoid conjugate vaccines, so it is difficult to assess the clinical significance of the difference in titers at the nine and 15 months age groups. Currently, either option could be pursued based on feasibility. However, longer-term effectiveness and duration of immunity studies will be critical to determine if immunogenicity wanes at different rates and if booster doses will be needed for either age group to achieve maximum protection throughout childhood.

TCV did not interfere with YF or MR immunogenicity in our study population. YF immunogenicity results are comparable to the immunogenicity generated by the YF vaccine when administered during routine EPI visits (Gotuzzo et al., 2013), or when co-administered with Vi polysaccharide typhoid vaccines to travelers (Alberer et al., 2015). While children may not respond as effectively as adults to YF immunization or may lose vaccine-induced immunity more rapidly than adults (Gotuzzo et al., 2013), the protection conferred by vaccination at an early age is essential for children living in endemic areas (Diseases and Injuries, 2020). Similarly, TCV did not interfere with MR immunogenicity. In our study population, 88·3% of participants achieved seropositivity for anti-measles antibody, which is consistent with results of previous studies that evaluated MR immunogenicity in other countries when administered as measles, mumps, rubella (MMR) vaccine with or without varicella vaccine (Ma et al., 2015). Measles is a global concern and a leading cause of death in young children. This study provides strong evidence that TCV can be safely and effectively co-administered with YF and MR vaccines without diminishing their substantial impact on child health.

The duration of follow-up is an important limitation of this study. Although safety follow-up for six months may be adequate to detect critical events related to vaccination, immune responses were only measured 28 days after vaccination. Therefore, studies documenting long-term immunity in this population are necessary to assess the protective effect of TCV more comprehensively.

Our study in Burkina Faso – the first to assess co-administration with yellow fever vaccine and the first to assess TCV at the 9-month vaccination visit in West Africa – is essential to inform country decisions on optimal TCV delivery strategies. To accommodate new vaccine introduction, adding visits to the current EPI schedule is not desirable and requires significantly more healthcare resources that may decrease compliance with the existing EPI schedule. Therefore, it is preferable to introduce new vaccines to routine visits. Evidence that TCV does not interfere with MR and YF vaccines routinely given at the 9-month visit in Burkina Faso provides the much-needed data to reassure public health authorities and medical providers that TCV can be added to this visit without diminishing current impacts. These data also show tolerability, safety, and immunogenicity of TCV in African children that are similar to children in other endemic areas. Future studies will address the important issue of the duration of vaccine-induced immunity against typhoid fever.

## Data sharing

After publication, the authors will provide participant data that underlie the results reported in this article, following de-identification (text, tables, figures, and appendices), to researchers who provide a methodologically sound proposal with approved aims. The study protocol will also be made available. Proposals should be directed to mlaurens@som.umaryland.edu; data requestors will need to sign a data access agreement to gain access. Proposals may be submitted up to 36 months following article publication. After 36 months, the data will be available in our university’s data warehouse, but without investigator support other than deposited metadata.

## Declaration of interests

The authors have no competing interests to declare.

## Contributions

KMN, SBS, MBL, and ETR conceived the study, developed protocol and SOPs, and managed ethical submissions. AO, NB, MS, AT, AK, ECB, and GDB recruited participants and performed participant follow-up procedures. INO, AD, and MO collected and processed clinical specimens for immunogenicity and generated anti-Vi and anti-tetanus titers. JJO and MFP generated anti-measles, anti-rubella, and anti-yellow fever titers. IS and AH managed study vaccines, performed randomization, and supervised injections. JKT designed and implemented REDCap data capture and management procedures to support the trial. YL developed a statistical analysis plan and conducted analyses. SD conducted analyses and created figures and tables. LPJ created figures and tables and edited the manuscript. All authors read and approved the final manuscript.

## Declaration of Competing Interest

The authors report no declarations of interest.
