# Supplementary material for: Safety and immunogenicity of Vi-typhoid conjugate vaccine co-administration with routine 9-month vaccination in Burkina Faso: A randomized controlled phase 2 trial
Source: Int J Infect Dis. 2021 Jul;108:465–72. doi: 10.1016/j.ijid.2021.05.061 (PMC8298254; doi:10.1016/j.ijid.2021.05.061)
Supplement: Supplementary file 1 [file mmc1.docx]

**Supplementary Appendix:**

|  |  | Male | | Female | |  |
| --- | --- | --- | --- | --- | --- | --- |
|  |  | **n** | **GMT (95% CI)** | **n** | **GMT (95% CI)** | **p-value*** |
| Group 1: TCV | Day 0 | 24 | 7·2 (4·3-11·9) | 25 | 11·0 (5·8- 20·7) | 0·34 |
|  | Day 28 | 24 | 860·4 (398·6-1857·3) | 25 | 1661·6 (994·8-2775·3) | 0·40 |
| Group 2: IPV | Day0 | 25 | 8·5 (4·7-15·3) | 26 | 8·4 (4·8-14·6) | 0·96 |
|  | Day 28 | 24 | 6·7 (4·5-9·8) | 26 | 11·7 (6·3-21·6) | 0·44 |
| n=number of participants. GMT=geometric mean titer. CI=confidence interval. GMT in ELISA (EU)/mL. | | | | | | |
| *Comparison of GMT between males and females using two-sample t-test on log_10_ transformed data. | | | | | | |

**Table A1 Anti-Vi IgG antibody geometric mean titers before vaccination (day 0) and 28 days after vaccination, by sex**

|  | **Group 1: TCV** | | **Group 2: IPV** | |
| --- | --- | --- | --- | --- |
|  | **n/N** | **% (95% CI)** | **n/N** | **% (95% CI)** |
| Short-term immunity |  |  |  |  |
| (>0·1 IU/mL) |  |  |  |  |
| Day 0 | 48/49 | 98·0 (89·2-100·0) | 51/51 | 100·0 (93·0-100·0) |
| Day 28 | 49/49 | 100·0 (92·8-100·0) | 50/50 | 100·0 (92·9-100·0) |
| Long-term immunity |  |  |  |  |
| (>1·0 IU/mL) |  |  |  |  |
| Day 0 | 28/49 | 57·1 (42·2-71·2) | 33/51 | 64·7 (50·1-77·6) |
| Day 28 | 48/49 | 98·0 (89·2-100·0) | 30/50 | 60·0 (45·2-73·6) |
| n=number of participants. N=total number. CI=confidence interval. IU=international unit. | | | | |

**Table A2 anti-tetanus IgG antibody immunogenicity before vaccination (day 0) and 28 days after vaccination**
